# Bi/CeO_2_–Decorated CuS Electrocatalysts for CO_2_-to-Formate Conversion

**DOI:** 10.3390/molecules29132948

**Published:** 2024-06-21

**Authors:** Qi Wang, Tianshuang Bao, Xiangchuan Zhao, Yue Cao, Jun Cao, Qiaoling Li, Weimeng Si

**Affiliations:** School of Materials Science and Engineering, Shandong University of Technology, Xincunxi Road 266th, Zibo 255000, China; wangqi199711@sina.com (Q.W.); baotianshuang@sina.com (T.B.); 17864301031@163.com (X.Z.); cao-yue@foxmail.com (Y.C.); juncao@sdut.edu.cn (J.C.)

**Keywords:** CO_2_RR, formate, Bi/CeO_2_–decorated CuS catalysts

## Abstract

The electrocatalytic carbon dioxide (CO_2_) reduction reaction (CO_2_RR) is extensively regarded as a promising strategy to reach carbon neutralization. Copper sulfide (CuS) has been widely studied for its ability to produce C_1_ products with high selectivity. However, challenges still remain owing to the poor selectivity of formate. Here, a Bi/CeO_2_/CuS composite was synthesized using a simple solvothermal method. Bi/CeO_2_–decorated CuS possessed high formate selectivity, with the Faraday efficiency and current density reaching 88% and 17 mA cm^−2^, respectively, in an H-cell. The Bi/CeO_2_/CuS structure significantly reduces the energy barrier formed by OCHO*, resulting in the high activity and selectivity of the CO_2_ conversion to formate. Ce^4+^ readily undergoes reduction to Ce^3+^, allowing the formation of a conductive network of Ce^4+^/Ce^3+^. This network facilitates electron transfer, stabilizes the Cu^+^ species, and enhances the adsorption and activation of CO_2_. Furthermore, sulfur catalyzes the OCHO* transformation to formate. This work describes a highly efficient catalyst for CO_2_ to formate, which will aid in catalyst design for CO_2_RR to target products.

## 1. Introduction

In recent decades, the utilization of fossil fuels as energy sources has resulted in a notable rise in the concentration of carbon dioxide (CO_2_) in the atmosphere. This increase poses a threat to the sustainable development of societies [1]. The electrocatalytic CO_2_ reduction reaction (CO_2_RR) has emerged as a promising approach for transforming CO_2_ into valuable products using electricity derived from renewable energy sources [2,3,4]. This electrochemical process involves multielectron/proton transfer mechanisms to convert CO_2_ into various chemicals, such as CO, HCOOH, CH_4_, CH_3_OH, C_2_H_4_, CH_3_COOH, and CH_3_CH_2_OH [5,6,7,8]. In these processes, the reduction to formic acid, which is usually considered an economically viable and atom-economic target, only requires two proton-coupled electron transfers [9]. Metals, like Zn, Sn, Bi, Pb, Au, and Ag, that have weaker binding capacities for CO intermediates may produce CO or HCOOH [10,11]. In general, the formation of the product involves three steps: (1) the adsorption of reactants on the surface of the electrocatalyst; (2) the proton-coupled electron transfer to the reactants; and (3) the desorption of electrocatalyst surface products [12,13,14]. At present, there are three pathways for CO_2_ to generate HCOOH that proceed via *OCHO, *COOH, and *H intermediates, where * represents the holes on the catalyst surface or adsorption intermediates [15]. Koh et al. showed using theoretical calculations that the *OCHO pathway is more energetically favorable on a bismuth (Bi) surface [16]. First, the CO_2_^−^ radical anion is formed by single-electron transfer to CO_2_ in which the oxygen in the CO_2_^−^ radical anion binds to the electrode surface [17]. In this case, protonation occurs on the carbon atom and the HCOO* intermediate forms. Then, the *OCHO intermediate is formed by electron transfer. Finally, HCOOH is generated by the *OCHO protonation step [12,18]. Bi–based catalysts have attracted attention owing to their low toxicity and cost [19]. However, the high overpotential for CO_2_RR to HCOOH and poor electronic conductivity hinder the electrocatalytic performance for the selective generation of HCOOH. In addition, owing to the inevitable complex reaction pathways and inherent low efficiency, the activity and selectivity of Bi–based electrocatalysts need to be further improved [20]. The addition of Bi on other metal substrates improves the stability of the catalysts [21]. Additionally, the introduction of a second metal regulates the electronic structure of the catalyst, thereby enhancing its selectivity, stability, and activity towards CO_2_RR. As a CO_2_RR catalyst, copper (Cu) has high electronic conductivity and tunable selectivity. Thus, Cu has been introduced into Bi to form bimetallic Bi–Cu materials that can enhance the conversion of CO_2_ to HCOOH [22]. These Cu–Bi alloys can be prepared by co-deposition, which expands the interfacial active area for CO_2_RR. The CuBi_3_ catalyst has an exceptional electrochemical performance for CO_2_RR towards HCOOH, with a Faraday efficiency (FE) of ~98.4% and a HCOOH partial current density of 21.2 mA cm^−2^ in 0.1 M KHCO_3_. The CuBi_3_ catalyst has the ability to regulate electronic states and has outstanding adsorption of CO_2_, providing a lattice and spatially constrained environment for active sites [23]. Copper sulfide (CuS) produces C_1_ products with high selectivity [24], and sulfur (S) can change the electronic properties of Cu, thereby influencing its capacity to adsorb pertinent intermediates like HCOO* [25]. Chen et al. prepared CuS catalysts with different morphologies and compositions by adjusting the Cu/S raw material ratio and the reaction temperature. In the solvothermal synthesis, the higher Cu/S ratio and reaction temperature leads to a relatively high selectivity of CuS nanoflowers for the production of HCOOH, with an FE of ~52% [26]. A Cu electrode with different oxidation states (Cu^+^ and Cu^0^-dominated) is capable of directing specific CO_2_RR pathways to generate HCOOH [27]. However, the in situ formation of Cu^+^ is insufficient and easily reduces to Cu^0^. Therefore, precisely controlling the local structure of the catalysts to generate stable and abundant Cu^+^ active sites is required to improve the activity and selectivity of the catalysts. Chen et al. reported that Cu^+^ is stabilized by the strong interaction between CuO and CeO_2_ for the highly selective electrocatalytic reduction of CO_2_ to ethylene under mild conditions. Adjusting the CuO/CeO_2_ interface interaction significantly inhibits proton reduction and enhances CO_2_ reduction. In 0.1-M KHCO_3,_ the ethylene FE is as high as 50.0% at −1.1 V vs. the reversible hydrogen electrode (RHE) [28]. Although previous studies on Cu–based catalysts have made great progress, it is still difficult to control the selectivity of CO_2_RR in Cu–based catalysts owing to the elusive influence of various entanglement factors on the complexity of the CO_2_RR pathway.

In this study, a simple strategy for the preparation of Bi– and CeO_2_–loaded CuS composites using the dissolution heat method is introduced. A synthesized Bi/CeO_2_/CuS catalyst was used to study the electrocatalytic performance of CO_2_RR to formate. Electrochemical tests demonstrated that Bi/CeO_2_/CuS effectively catalyzed the generation of formate from CO_2_, achieving a FE_formate_ of 88% and current density of 17 mA cm^−2^. The incorporation of CeO_2_ enhanced conductivity and stabilized Cu^+^, which promoted CO_2_ adsorption and activation. The CuS structure of the Bi coating protected the stable existence of S and prevented the air-based oxidation of Cu. This study provides an effective catalyst for the production of HCOOH through CO_2_RR and helps to promote catalyst designs for CO_2_RR target products.

## 2. Results and Discussion

**Figure 1:**
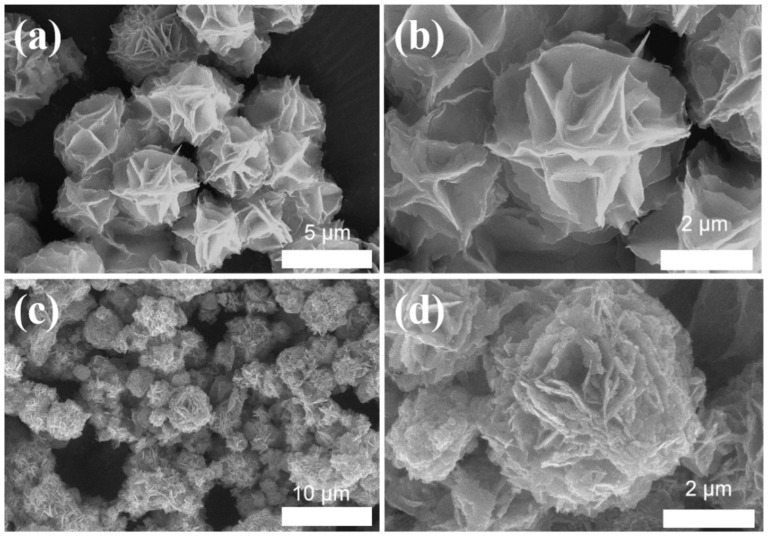
SEM images of (**a**,**b**) CuS and (**c**,**d**) Bi/CeO_2_/CuS.

Figure 1a,b present scanning electron microscope (SEM) images of CuS that display flower-like shapes. The nanosheets possessed smooth surfaces and had an interval range of 0.5–1 μm. A CuS catalyst loaded with Bi and CeO_2_, as shown in Figure 1c,d, revealed an uneven surface, and the nanosheets exhibited rough surfaces that are thicker at the edges. The uneven surface offered a high specific surface area. In addition, the nanoflower morphology provided more under-coordinated sites for CO_2_RR.

**Figure 2:**
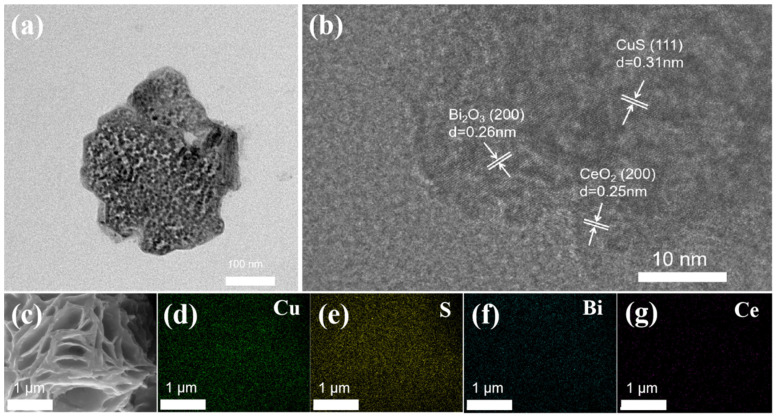
(**a**) TEM images of Bi/CeO_2_/CuS; (**b**) high-resolution TEM images of Bi/CeO_2_/CuS; and (**c**–**g**) EDS mapping of Bi/CeO_2_/CuS.

Figure 2a presents transmission electron microscope (TEM) images of a Bi/CeO_2_/CuS catalyst. There was material cover around the CuS. The lattice interplanar spacings of nanosheets measured 0.26 nm, 0.34 nm, and 0.31 nm, corresponding to the (200) plane of Bi_2_O_3_, the (111) plane of CuS, and the (111) plane of CeO_2_ [29,30], respectively (Figure 2b). CeO_2_ covered the surface of the CuS, providing oxygen to the neighboring Cu [31]. Figure 2c–g show the elemental distributions of Cu, S, and Ce. The distribution of CeO_2_ on the surface of the CuS nanosheets was uniform, and there was no observed particle agglomeration.

**Figure 3:**
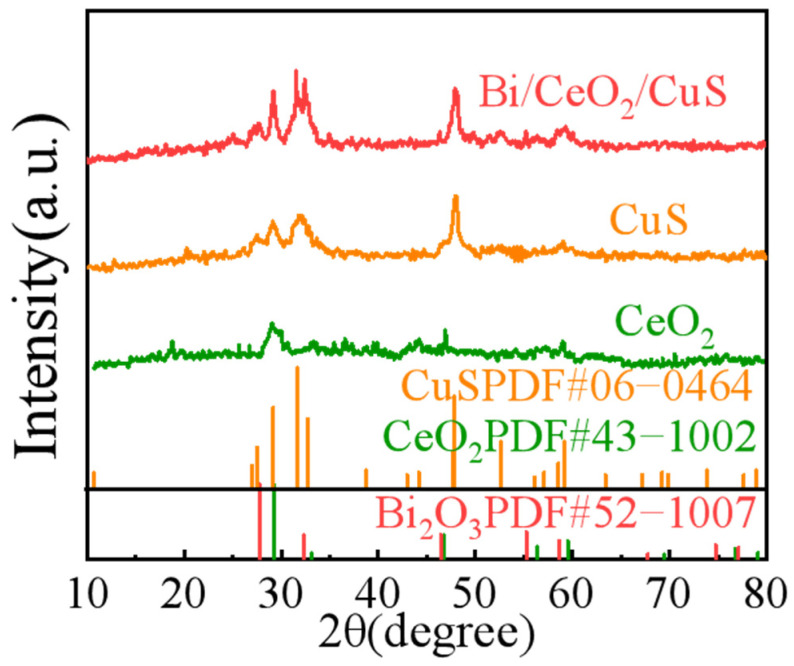
XRD patterns of Bi/CeO_2_/CuS, CuS, and CeO_2_.

The crystal structure of the sample was analyzed using X-ray diffraction (XRD), and the diffraction spectra (Figure 3) displayed distinctive peaks at the 2θ positions of 29.2°, 31.7°, 32.8°, and 47.9°, which corresponded to the CuS crystals (PDF#06–0464) [32]. The peaks at the 2θ positions of 29.2°, 33.07°, 46.7°, and 56.34° corresponded to the CeO_2_ crystals (PDF#43–1002). The XRD pattern of the Bi/CeO_2_/CuS catalyst revealed clear diffraction peaks of Bi_2_O_3_ and CeO_2_ [33,34]. These results indicated the successful preparation of Bi/CeO_2_/CuS.

**Figure 4:**
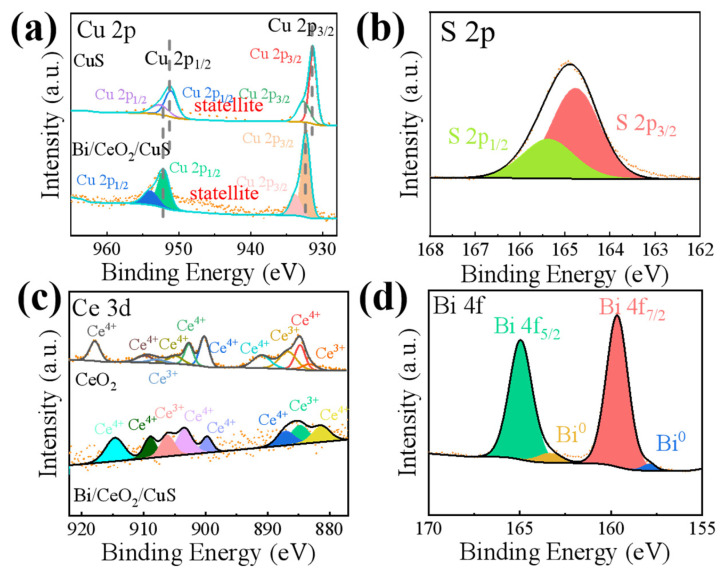
XPS patterns of Bi/CeO_2_/CuS (**a**) Cu 2p, (**b**) S 2p, (**c**) Ce 3d, and (**d**) Bi 4f.

The valence and electronic structures of the elements Cu, S, and Ce in the Bi/CeO_2_/CuS catalyst were investigated using XPS. In the Cu 2p spectrum (Figure 4a), two main peaks appeared at 932.38 eV and 952.23 eV, corresponding to Cu 2p_3/2_ and Cu 2p_1/2_, respectively. These could be divided into Cu^2+^2p_3/2_ (932.13 eV), Cu^2+^2p_3/2_ (933.83 eV), Cu^2+^2p_1/2_ (952.13 eV), and Cu^2+^2p_1/2_ (954.13 eV) [35]. The two subpeaks with binding energies located at ~931.58 and 951.28 eV indicated the presence of Cu–S bonds. The characteristic peaks at ~933.3 and 954.13 eV, as well as two satellite peaks at 945.23 eV and 963.08 eV, were attributed to Cu–O for the surface oxidation of Cu in air [28,36]. For Bi/CeO_2_/CuS, a positive shift of Cu 2p was observed, indicating that electron transfer occurred on the surface of the Cu [22]. The two peaks in the S 2p XPS spectrum (Figure 4b) with binding energies at 164.43 eV and 165.13 eV were attributed to S 2p_3/2_ and S 2p_1/2_, corresponding to metal sulfide [24]. In the Ce 3d spectrum, as shown in Figure 4c, the peaks of CeO_2_ at 881.23, 887.18, 899, 903.53, 909.03, and 914.53 eV were attributed to the mixed configurations of the 3d^9^4f^1^, 3d^9^4f^2^, and 3d^9^4f^0^ Ce^4+^ states. The peaks at 884.78 and 908.78 eV were attributed to the mixed configuration of the 3d^9^4f^1^ and 3d^9^4f^2^ Ce^3+^ states. Thus, CeO_2_ contained Ce^3+^ and Ce^4+^ [28]. In addition, the binding energies of the Ce elements in the Bi/CeO_2_/CuS composite were less than that of CeO_2_, indicating that CeO_2_ gained electrons, and the electron cloud density of CeO_2_ in the Bi/CeO_2_/CuS composite materials increased [37]. As shown in Figure 4c, the Bi 4f spectrum had four peaks at 157.88, 159.68, 163.28, and 165.03 eV. The first double peak at 157.88 and 163.28eV corresponded to the Bi 4f_7/2_ and 4f_5/2_ of Bi^0^ [38]. The second double peak at 159.68 and 165.03 eV was attributed to a layer of Bi_2_O_3_ [22].

**Figure 5:**
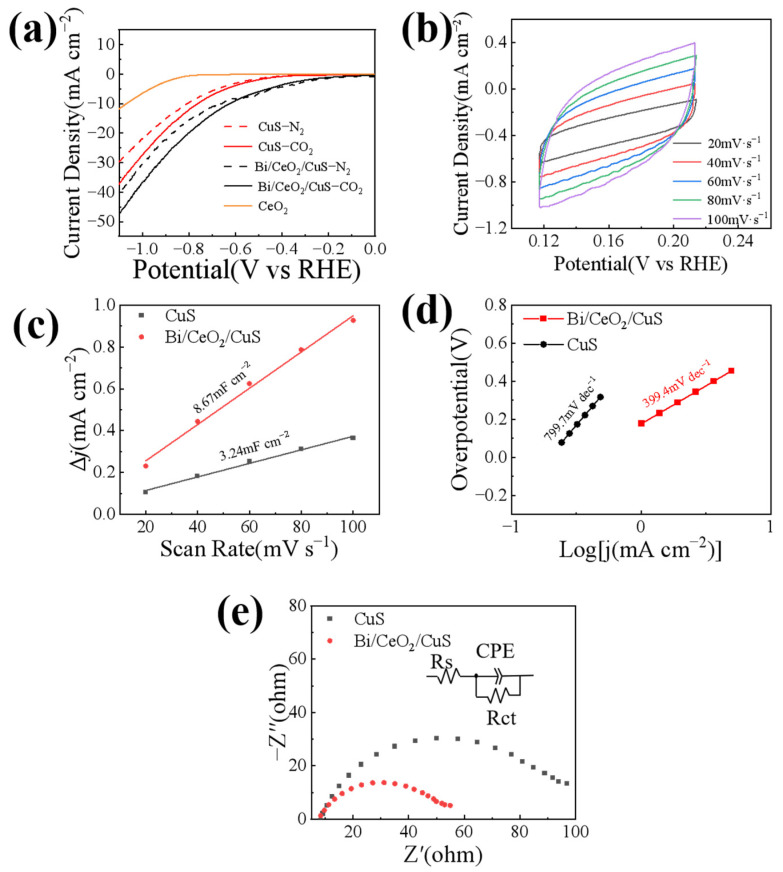
(**a**) LSV curves, (**b**) CV curves, (**c**) electrochemical active areas, (**d**) Tafel plots, and (**e**) electrochemical impedance spectra of Bi/CeO_2_/CuS and CuS.

The catalytic performances of Bi/CeO_2_/CuS were investigated in a sealed H-type electrolytic cell with a three-electrode system. The experiment utilized a CO_2_–saturated 0.5 M KHCO_3_ electrolyte. Figure 5a shows the linear sweep voltammetry (LSV) curves of CuS, CeO_2_, and Bi/CeO_2_/CuS in N_2_–saturated and CO_2_–saturated electrolytes with a scan rate of 10 mV s^−1^. For the lack of active sites, CeO_2_ does not respond to CO_2_. The cathodic current densities in the CO_2_–saturated electrolytes were higher than those in the N_2_–saturated electrolytes within the potential range of −0.2 V to −1.1 V vs. RHE. Additionally, the Bi/CeO_2_/CuS catalysts had lower onset potentials than the CuS in the CO_2_–saturated electrolytes. Furthermore, the current density of the Bi/CeO_2_/CuS was significantly higher than that of the CuS, suggesting that Bi/CeO_2_/CuS has a comparatively superior activity of CO_2_RR [22]. At a potential of −1.1 V vs. RHE, the current density of CeO_2_/CuS reached 47.3 mA cm^−2^, which was 1.3 times higher than that of the CuS. This enhancement was attributed to the reduction of Ce^4+^ to Ce^3+^ in CeO_2_, which created a conductive network (Ce^4+^/Ce^3+^) and improved the conductivity of the catalyst, enhancing the electrocatalytic performance of the CuS.

To further demonstrate the superior electrocatalytic performance of Bi/CeO_2_/CuS over CuS, CV curves were obtained at different scanning speeds (20, 40, 60, 80, and 100 mV s^−1^) (Figure 5b), and the electrochemical active area of the sample was evaluated by calculating the double-layer capacitance (C_dl_). As shown in Figure 5c, the C_dl_ (8.67 mF cm^−2^) of Bi/CeO_2_/CuS was greater than that of CuS (3.24 mF cm^−2^). The addition of CeO_2_ effectively increased the active area of the reaction, and the unique structure of Bi/CeO_2_/CuS resulted in good electrocatalytic activity. To reveal the mechanism behind the performance enhancement, the reaction kinetics of the catalyst in the electrocatalytic reduction of CO_2_ were studied. The Tafel slope was obtained by fitting the LSV curve to evaluate different catalysts’ CO_2_RR kinetic performances. As shown in Figure 5d, the Tafel slope of Bi/CeO_2_/CuS (399.4 mV dec^−1^) was much smaller than that of CuS (799.7 mV dec^−1^), indicating that Bi/CeO_2_/CuS is more prone to generate *CO_2_^−^ intermediates compared with CuS [39,40]. In a further comparison of the conductivity of the CuS and Bi/CeO_2_/CuS catalysts, the electrochemical impedance spectra of CuS and Bi/CeO_2_/CuS were determined in 0.1-M CO_2_–saturated KHCO_3_ solution at −0.9 V vs. RHE. As shown in Figure 5e, the Bi/CeO_2_/CuS exhibited an obviously smaller equivalent series resistance than that of CuS, illustrating its faster catalytic kinetics [41]. In addition, chronocoulometry was used to evaluate the electrochemically effective area of the catalysts (Figure S5), which were 0.0132 cm^2^ and 0.032 cm^2^ for CuS and Bi/CeO_2_/CuS, respectively. This indicated that the Bi/CeO_2_/CuS catalyst possessed a high electroactive surface area, which enhanced the electrochemical response [42].

**Figure 6:**
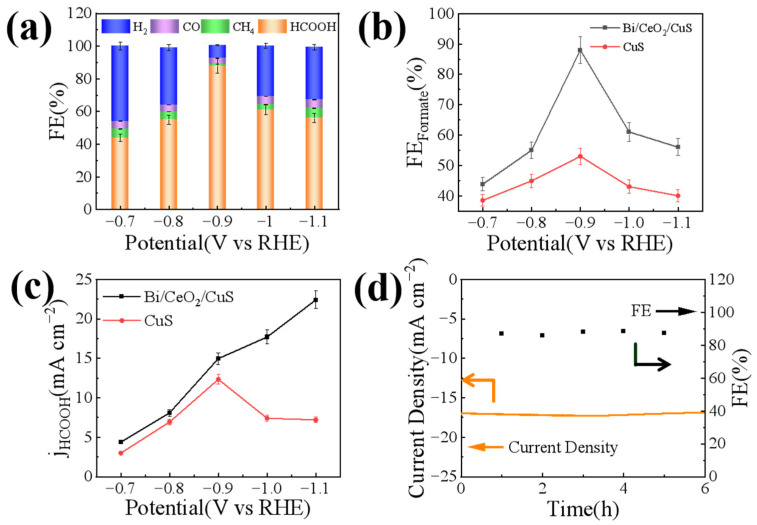
(**a**) FEs of each product of Bi/CeO_2_/CuS, (**b**) FEs of formate for Bi/CeO_2_/CuS and CuS, (**c**) HCOOH partial current densities of Bi/CeO_2_/CuS and CuS, and (**d**) durability test of Bi/CeO_2_/CuS.

The electrocatalytic reduction of CO_2_ by Bi/CeO_2_/CuS was investigated at various constant potentials using the constant potential method. The liquid product obtained from electrocatalytic CO_2_ production was found to be pure formate (Figure 6a and Figure S3). Figure 6a shows the FEs for formate production from the catalysts at different potentials, with Bi/CeO_2_/CuS exhibiting the highest FE for formate at all potentials. The maximum FE of formate reached 88% at −0.9 V vs. RHE. Compared with the Bi/CeO_2_/CuS catalyst, the FE_HCOOH_ of CuS was significantly lower. These results indicated that Bi/CeO_2_/CuS had the highest electrochemical reduction activity for CO_2_. Moreover, the FE of Bi/CeO_2_/CuS was higher than that of CuS, suggesting that Bi/CeO_2_/CuS is more suitable for the electrochemical reduction of CO_2_ and that it exhibits higher selectivity towards formate. This can be attributed to the S atom energy in the Cu lattice, which effectively reduces the Gibbs free energy for the conversion of CO_2_ into *OCHO intermediates. These intermediates play crucial roles in the formation of formate. Additionally, Bi doping is conducive to the adsorption and activation of CO_2_ and accelerated electron transfer processes [43]. Therefore, Bi/CeO_2_/CuS demonstrates the capability to generate formate with high selectivity [44,45]. Ce^4+^ in CeO_2_, which is easily reduced at a negative potential [46], stabilizes Cu^+^ species. This increases the adsorption and activation of CO_2_, as well as the conversion of HCOO* intermediates, which further improves the FE of formate. Figure 6b shows the FE of formate at different potentials, with a potential window of −0.4 V to −0.9 V vs. RHE for the generation of formate for Cu–based catalysts [47]. The FE of formate decreased when the applied constant potential was more negative than −0.9 V vs. RHE, which was caused by transport limitations [48]. The formate partial current densities of the catalysts at different point positions were calculated and compared. As shown in Figure 6c, the formate partial current density of Bi/CeO_2_/CuS was higher than that of CuS, showing an increasing trend from −0.8 V to −1.05 V vs. RHE. To further verify the stability of Bi/CeO_2_/CuS in the electrocatalytic reduction of CO_2_, a constant potential test was carried out at a potential of −0.9 V vs. RHE (Figure 6d). The Bi/CeO_2_/CuS catalyst exhibited an apparently stabilized FE_HCOOH_ and current density at −0.9 V vs. RHE in CO_2_–saturated 0.5-M KHCO_3_ for over 6 h. Meanwhile, the FE_HCOOH_ remained above 85%.

**Figure 7:**
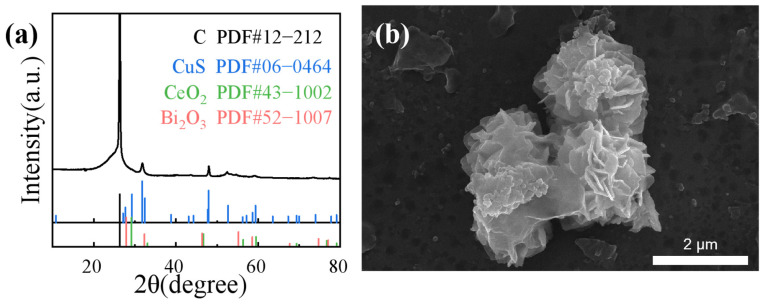
(**a**) XRD pattern and (**b**) SEM image of Bi/CeO_2_/CuS after CO_2_RR.

The structural stability of Bi/CeO_2_/CuS after CO_2_RR at −0.9 V vs. RHE was characterized by XRD and SEM. As shown in Figure 7a, the obvious diffraction peak belonged to carbon paper. Other peaks correspond to the main peak position of Bi/CeO_2_/CuS (29.2–32.8°, 46.7–48.2°, 55.2°, 58.6–59.5°). Affected by the C peak, the peak resolution of Bi/CeO_2_/CuS is reduced, merging into a typical broad peak. Figure 7b show the SEM images after the reaction. The morphology remains uniform particles, similar to the morphology before the reaction. Based on the XRD and SEM results, we believe that the catalysts are relatively stable. Comparing with the previous results in Table 1, it can be seen the Bi/CeO_2_/CuS fabricated in this research has good performance as a CO_2_ electrochemical catalysts.

**Figure 8:**
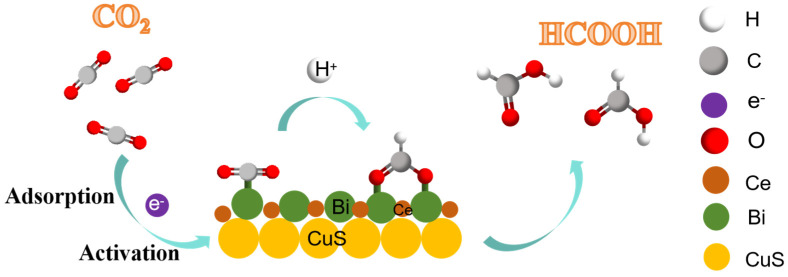
Schematic diagram of the possible reaction pathways of the CO_2_RR to HCOOH.

The possible reaction pathway of CO_2_RR to HCOOH over the Bi/CeO_2_/CuS catalyst is shown in Figure 8. The CO_2_ was adsorbed by the catalyst, and it captured an electron and proton to form the *COOH intermediate, which was further converted into HCOOH with the help of electrons. In addition, the HCOO* intermediate produced HCOOH [25]. Specifically, the adsorbed CO_2_ was activated to form *CO_2_, the *CO_2_ was protonated to form the HCOO* intermediate, and proton–electron transfer formed HCOOH [49]. The mechanism is as follows [24,25]:

CO_2_ + e^−^ → *CO_2_^−^ (1)

*CO_2_^−^ + H^+^ → *OCHO (2)

*OCHO + e^−^ + H^+^ → HCOOH. (3)

The HCOO* intermediate generated HCOOH on Cu (111) through proton–electron pair transfer (formate binds to the surface through its O atom) [14]. The doping of S into Cu (111) affects the formation of HCOOH. The presence of S weakens the adsorption and desorption of HCOO* and *COOH, and inhibits the formation of CO [24]. Additionally, the presence of S species facilitates the formation of the key *OCHO intermediates toward HCOOH production [50]. Furthermore, the incorporation of S into the Cu surface lowers the performance of the Cu catalyst for H_2_ evolution [51]. However, the addition of CeO_2_ forms a Ce^3+^/Ce^4+^ conductive network, which improves the catalyst’s conductivity [46], reduces the electron density of the Cu^2+^ site, and changes the valence state of Cu at the Bi/CeO_2_/CuS interface [28]. The Bi nanoparticles provide active sites and facilitate electron transfer.

**Table 1:** Comparison of FE_HCOOH_ for Bi/CeO_2_/CuS electrode with reported Cu–based electrocatalysts.

Catalysts	Cell Type	Electrolyte	FE(%)	Current Density (mA cm^−2^)	Ref.
Cu-xS	H-cell	0.1 M KHCO_3_	74	13.9	[52]
CuSx	H-cell	0.1 M KHCO_3_	75	12	[24]
Cu_7_S_4_ NSs	Flow cell	1 M KOH	82.7	456	[53]
GDY/CuSx	H-cell	0.1 M KHCO_3_	70	65.6	[54]
CuS/N,S-rGO	H-cell	0.5 M KHCO_3_	82	24.2	[36]
Sn–Cu@Sn	GDE	1 M KHCO_3_	84.2	30	[55]
CuBi_3_	H-cell	0.1 M KHCO_3_	98.4	21.2	[23]
Bi@NCA	H-cell	0.5 M KHCO_3_	95	–	[43]
Bi-PVP/CC-600	H-cell	0.5 M KHCO_3_	81	54	[56]
Bi-NFs	H-cell	0.1 M KHCO_3_	92.3	28.5	[41]
Bi/CeO_2_/CuS	H-cell	0.5 M KHCO_3_	88	17	This work

Cu-xS: Sulfur-doped Cu_2_O–derived Cu catalyst; CuSx: Sulfur-doped Cu catalyst; Cu_7_S_4_ NSs: Cu_7_S_4_ nanosheets; GDY/CuSx: Graphdiyne/copper sulfide heterostructure; CuS/N,S-rGO: CuS anchored on nitrogen and sulfur Co-doped graphene; Sn–Cu@Sn: Sn–Cu@Sn dendrites that have a core@shell architecture. CuBi_3_: Co-deposition to prepare Cu–Bi alloy. Bi@NCA: Bi nanoparticles were anchored on N–doped carbon aerogel. Bi-PVP/CC-600: Ultrasmall Bi nanoparticles confined in carbon nanosheets. Bi-NFs: Bi nanoflowers.

## 3. Experimental Section

### 3.1. Materials

Copper chloride dihydrate (CuCl_2_·2H_2_O, AR, 99%), thiourea (CH_4_N_2_S, AR, 99%), ethylene glycol (C_2_H_6_O_2_, AR, 98%), cerium (III) nitrate hexahydrate (Ce(NO_3_)_3_·6H_2_O, 99.9%), bismuth (III) nitrate hexahydrate (Bi(NO_3_)_3_·5H_2_O, AR.), nafion (5 wt.%), potassium bicarbonate (KHCO_3_), dimethyl sulfoxide (C_2_H_6_OS), deuterium oxide (D_2_O), and absolute ethanol were purchased from Macklin, Shanghai, China. CO_2_ (99.999%) was purchased from Shandong Baiyan Chemical Co., Ltd., Zibo, China. Carbonized paper (hydrophobic) was purchased from Shanghai Hesen Electric Co., Ltd., Shanghai, China.

### 3.2. Catalyst Preparation

#### 3.2.1. Synthesis of CuS Nanosheets

The CuS nanosheets were prepared in accordance with the solvothermal method [50]. Briefly, 0.85 g CuCl_2_·2H_2_O was dissolved in 30 mL ethylene glycol and stirred for 30 min in water bath at 90 °C. The mixture was stirred to obtain a uniform solution. Then, 1.52 g thiourea dissolved in 25 mL ethylene glycol was added slowly into the CuCl_2_·2H_2_O solution, and stirring continued for 30 min. The mixture was transferred to 100 mL polytetrafluoroethylene-lined stainless steel high-pressure reactor at 170 °C for 5 h. Finally, the obtained black product was centrifuged and washed several times with absolute ethanol and deionized water. It was then dried in a vacuum oven at 60 °C for 6 h to obtain CuS nanosheets.

#### 3.2.2. Synthesis of Bi/CeO_2_/CuS Nanosheets

Briefly, 100 mg of CuS nanosheets was dispersed into 40 mL of deionized water and stirred for 1 h. Subsequently, 20 mL 0.023 mol L^−1^ Ce(NO_3_)_3_ of ethanol solution was dropped into the CuS dispersion with continuous stirring. Then, 200 mg of Bi(NO_3_)_3_·5H_2_O was added to the solution. The mixture then underwent hydrothermal synthesis at 170 °C for 5 h. Finally, the obtained black product was centrifuged and washed several times with absolute ethanol and deionized water. Then, the obtained powder was dried at 60 °C for 6 h in vacuum oven to obtain Bi/CeO_2_/CuS nanosheets.

#### 3.2.3. Preparation of Working Electrodes

To construct the cathode electrode, 10 mg of powder prepared above was suspended in 1 mL acetone supplemented with 20 μL Nafion (5 wt%) via ultrasound. Then, the catalyst slurry (0.1 mL) was slowly drop cast onto a PTFE–hydrophobized carbon fiber paper (1 cm × 1 cm) to achieve a catalyst loading of ~1.0 mg cm^−2^.

### 3.3. Catalyst Characterization

The crystal structure of the catalyst was analyzed using an X-ray diffractometer from Bruker AXS (Billerica, MA, USA), using Cu Kα radiation in the wide 2θ range of 10–90°. The morphology and elemental mapping images of the catalysts were characterized using an FEI Sirion 200 field emission SEM (Portland, OR, USA). TEM images were recorded on an FEI Tecnai F20 TEM (Portland, OR, USA) at an operating voltage of 200 KV. XPS spectra were recorded on a K-Alpha (Thermo Fisher Scientific Co., Ltd., Waltham, MA, USA) instrument. The liquid product was quantitatively analyzed using a Bruker AVANCE 400 MHz (Billerica, MA, USA) nuclear magnetic resonance spectrometer. A gas chromatograph (Shandong Lunan Ruihong Chemical Instrument Co., Ltd.; SP-7890 Plus, Tengzhou, China) was used for the quantitative analysis of gas products.

### 3.4. CO_2_RR Performance

The CO_2_RR performance of the catalyst was tested using the three-electrode system of CS-350 electrochemical workstation (Wuhan Correst Instrument Co., Ltd., Wuhan, China). The experiment was performed using a sealed H-type electrolytic cell, and an air tightness test was conducted prior to the experiment. The working electrode consisted of catalyst-loaded carbon paper, an Ag/AgCl reference electrode, and a carbon rod counter electrode. A proton exchange membrane was utilized to separate the cathode chamber and the anode chamber, and a 0.5-M KHCO_3_ solution was employed as the electrolyte. Before each test, continuously bubbling high-purity CO_2_ (at a rate of 20 mL min^−1^) into the electrolyte for 30 min is recommended. Cyclic voltammetry scans at a scanning rate of 100 mV s^−1^ to activate and stabilize the electrode material were performed. LSV was conducted at a scanning rate of 10 mV s^−1^ in N_2_–saturated and CO_2_–saturated 0.5-M KHCO_3_ solution with a voltage range from 0 to −1.1 V (vs. RHE). The potentiostatic method was used to test the Faraday efficiency of catalysts, with a potential range of −0.7 V~−1.1V (vs. RHE). The Tafel slope (b) was obtained by fitting the linear portion of the Tafel plot to the Tafel equation (η = b lgJ + a) [57]. The test voltage for electrochemical impedance test was −0.1 V, test frequency 105–0.01 Hz, and amplitude 5 mV. Double-layer capacitance was determined by CV with different scan rates from 20 to 100 mV s^−1^ [58]. All of the electrochemical tests were performed without iR compensation.

### 3.5. Product Analysis

After electrolysis at a constant potential for 60 min, the gas products were collected using a gas collection bag and analyzed using a gas chromatograph equipped with a hydrogen flame ionization detector for CO and CH_4_ or a thermal conductivity detector for H_2_. The concentrations of the gas products were calculated from the ratio of the peak areas of the gas products and the standard gas. The FEs of the gas products were reported as the averages of three measurements.

Liquid products were analyzed by nuclear magnetic resonance spectrometry [59]. To quantify the concentration of formate, the concentration of 0.47-M dimethyl sulfoxide solution was used as the internal standard. A calibration curve was prepared using the NMR peak area of a standard concentration of HCOONa solution relative to the internal standard. Subsequently, the concentration of formate was determined by measuring the NMR peak area of formate relative to the internal standard, using the calibration curve as a reference. This approach enabled the accurate quantification of the formate concentration.

The FE was calculated as follows [60]:

FE (%) = (*x* × *n* × *F*/*Q*) × 100%, (4)

where *x* represents the molar amount of the product produced (mol) and *n* represents the number of electrons transferred to form different products. For example, in the case of formate, *n* is 2. *F* represents the Faraday constant (96,485 C mol^−1^), and *Q* represents the total amount of electricity consumed throughout the reaction (C).

The FE of gas products was calculated as follows:

FE (%) = (*N* × *Vp* × *Cst* × *P* × *F*)/(*Vst* × *R* × *T*× *j*× *t*), (5)

where *N* represents the number of electrons transferred to form gas molecules, *Vp* represents the peak area of gas products in GC spectrum, *Cst* represents the volume concentration of standard gas, *P* represents the standard atmospheric pressure (101.3 kpa), *F* represents the Faraday constant (96,485 C mol^−1^), *Vst* represents the peak area of standard gas in GC spectrum, *T* represents the room temperature (298 K), *j* represents the recorded current, and *t* represents the reaction time.

## 4. Conclusions

In this work, we demonstrated that Bi/CeO_2_/CuS can be used as a highly active and selective catalyst for the electrochemical reduction of CO_2_ to generate formate in a wide potential range. At −0.9 V vs. RHE, the FE of formate reached 88% and the current density was −17 mA cm^−2^. Bi/CeO_2_/CuS also showed excellent stability. After a 1 h reaction, FE of formate remained stable at −0.9 V vs. RHE, as did the structure. The S inhibited hydrogen evolution reactions, CeO_2_ improved the catalyst conductivity, and Bi provided active sites and facilitated electron transfer. This work provides an easily synthesizable catalyst for the generation of formate by electrocatalytic CO_2_ reduction, and these findings will aid in the rational design of Cu–based catalysts for the electroreduction of CO_2_.

## Data Availability

Data are contained within the article or Supplementary Materials. The data sets generated during and/or analyzed during the current study are available from the corresponding author upon reasonable request.

## Supplementary Materials

The following supporting information can be downloaded at: https://www.mdpi.com/article/10.3390/molecules29132948/s1, Reference [41] are cited in the supplementary materials. Figure S1. Survey of Bi/CeO_2_/CuS. Figure S2. CV curve of CuS. Figure S3. The FE for all products of CuS catalyst. Figure S4. The current density of Bi/CeO_2_/CuS at different potential. Figure S5. The effective surface area curves of different simples. Figure S6. Standard curve of formate. Figure S7. Formate yield of Bi/CeO_2_/CuS. Figure S8. (a–c) The NMR of different concentration standard, (d) The NMR of Bi/CeO_2_/CuS at −0.9 V vs. RHE. Figure S9. (a) The GC of standard, (b) The GC FID 1 image of simple, (c) The GC FID 2 image of simple, (d) The GC TCD image of simple.

## References

[1] Zickfeld K., MacDougall A.H., Matthews H.D. (2016). On the proportionality between global temperature change and cumulative CO_2_ emissions during periods of net negative CO_2_ emissions. Environ. Res. Lett..

[2] Chen H., Fu W., Geng Z., Zeng J., Yang B. (2021). Inductive effect as a universal concept to design efficient catalysts for CO_2_ electrochemical reduction: Electronegativity difference makes a difference. J. Mater. Chem. A.

[3] Zhu P., Wang H. (2021). High-purity and high-concentration liquid fuels through CO_2_ electroreduction. Nat. Catal..

[4] Bagger A., Ju W., Varela A.S., Strasser P., Rossmeisl J. (2019). Electrochemical CO_2_ reduction: Classifying Cu facets. ACS Catal..

[5] Chen H., Wang Z., Wei X., Liu S., Guo P., Han P., Wang H., Zhang J., Lu X., Wei B. (2021). Promotion of electrochemical CO_2_ reduction to ethylene on phosphorus-doped copper nanocrystals with stable Cu^δ+^ sites. Appl. Surf. Sci..

[6] Zhang Y., Jiang H., Niu D., Manke I., Yang C., Zhu M., Zhang X., Chen R. (2022). Pyridine-grafted nitrogen-doped carbon nanotubes achieving efficient electroreduction of CO_2_ to CO within a wide electrochemical window. J. Mater. Chem. A.

[7] Lin L., He X., Zhang X.G., Ma W., Zhang B., Wei D., Xie S., Zhang Q., Yi X., Wang Y. (2023). A nanocomposite of bismuth clusters and Bi_2_O_2_CO_3_ sheets for highly efficient electrocatalytic reduction of CO_2_ to formate. Angew. Chem. Int. Ed..

[8] Zheng T., Zhang M., Wu L., Guo S., Liu X., Zhao J., Xue W., Li J., Liu C., Li X. (2022). Upcycling CO_2_ into energy-rich long-chain compounds via electrochemical and metabolic engineering. Nat. Catal..

[9] Verma S., Kim B., Jhong H.R.M., Ma S., Kenis P.J. (2016). A gross-margin model for defining technoeconomic benchmarks in the electroreduction of CO_2_. ChemSusChem.

[10] Seong H., Efremov V., Park G., Kim H., Yoo J.S., Lee D. (2021). Atomically precise gold nanoclusters as model catalysts for identifying active sites for electroreduction of CO_2_. Angew. Chem. Int. Ed..

[11] Lim C., Lee W.H., Won J.H., Ko Y.J., Kim S., Min B.K., Lee K.Y., Jung W.S., Oh H.S. (2021). Enhancement of catalytic activity and selectivity for the gaseous electroreduction of CO_2_ to CO: Guidelines for the selection of carbon supports. Adv. Sustain. Syst..

[12] Zhao S., Li S., Guo T., Zhang S., Wang J., Wu Y., Chen Y. (2019). Advances in Sn-Based Catalysts for Electrochemical CO_2_ Reduction. Nano-Micro Lett..

[13] Peterson A.A., Abild-Pedersen F., Studt F., Rossmeisl J., Nørskov J.K. (2010). How copper catalyzes the electroreduction of carbon dioxide into hydrocarbon fuels. Energy Environ. Sci..

[14] Yoo J.S., Christensen R., Vegge T., Nørskov J.K., Studt F. (2016). Theoretical Insight into the Trends that Guide the Electrochemical Reduction of Carbon Dioxide to Formic Acid. ChemSusChem.

[15] Guo S., Liu Y., Murphy E., Ly A., Xu M., Matanovic I., Pan X., Atanassov P. (2022). Robust palladium hydride catalyst for electrocatalytic formate formation with high CO tolerance. Appl. Catal. B Environ..

[16] Koh J.H., Won D.H., Eom T., Kim N.-K., Jung K.D., Kim H., Hwang Y.J., Min B.K. (2017). Facile CO_2_ Electro-Reduction to Formate via Oxygen Bidentate Intermediate Stabilized by High-Index Planes of Bi Dendrite Catalyst. ACS Catal..

[17] Feaster J.T., Shi C., Cave E.R., Hatsukade T., Abram D.N., Kuhl K.P., Hahn C., Nørskov J.K., Jaramillo T.F. (2017). Understanding Selectivity for the Electrochemical Reduction of Carbon Dioxide to Formic Acid and Carbon Monoxide on Metal Electrodes. ACS Catal..

[18] Birdja Y.Y., Pérez-Gallent E., Figueiredo M.C., Göttle A.J., Calle-Vallejo F., Koper M.T.M. (2019). Advances and challenges in understanding the electrocatalytic conversion of carbon dioxide to fuels. Nat. Energy.

[19] Wu M., Xu B., Zhang Y., Qi S., Ni W., Hu J., Ma J. (2020). Perspectives in emerging bismuth electrochemistry. Chem. Eng. J..

[20] Xue J., Fu X., Geng S., Wang K., Li Z., Li M. (2023). Boosting electrochemical CO_2_ reduction via valence state and oxygen vacancy controllable Bi–Sn/CeO_2_ nanorod. J. Environ. Manag..

[21] Peng L., Wang Y., Wang Y., Xu N., Lou W., Liu P., Cai D., Huang H., Qiao J. (2021). Separated growth of Bi-Cu bimetallic electrocatalysts on defective copper foam for highly converting CO_2_ to formate with alkaline anion-exchange membrane beyond KHCO_3_ electrolyte. Appl. Catal. B Environ..

[22] Dou T., Song D., Wang Y., Zhao X., Zhang F., Lei X. (2023). Hierarchical Bi/S-modified Cu/brass mesh used as structured highly performance catalyst for CO_2_ electroreduction to formate. Nano Res..

[23] Fu Y., Leng K., Zhuo H., Liu W., Liu L., Zhou G., Tang J. (2023). Nanoconfinement effects on CuBi_3_ alloy catalyst for efficient CO_2_ electroreduction to formic acid. J. CO_2_ Util..

[24] Deng Y., Huang Y., Ren D., Handoko A.D., Seh Z.W., Hirunsit P., Yeo B.S. (2018). On the Role of Sulfur for the Selective Electrochemical Reduction of CO_2_ to Formate on CuSx Catalysts. ACS Appl. Mater. Interfaces.

[25] Dou T., Qin Y., Zhang F., Lei X. (2021). CuS Nanosheet Arrays for Electrochemical CO_2_ Reduction with Surface Reconstruction and the Effect on Selective Formation of Formate. ACS Appl. Energy Mater..

[26] Chen J., Tu Y., Zou Y., Li X., Jiang J. (2021). Morphology and composition-controllable synthesis of copper sulfide nanocrystals for electrochemical reduction of CO_2_ to HCOOH. Mater. Lett..

[27] Wang J., Tan H.Y., Zhu Y., Chu H., Chen H.M. (2021). Linking the dynamic chemical state of catalysts with the product profile of electrocatalytic CO_2_ reduction. Angew. Chem. Int. Ed..

[28] Chu S., Yan X., Choi C., Hong S., Robertson A.W., Masa J., Han B., Jung Y., Sun Z. (2020). Stabilization of Cu^+^ by tuning a CuO–CeO_2_ interface for selective electrochemical CO_2_ reduction to ethylene. Green Chem..

[29] Liang Y., Wu C., Meng S., Lu Z., Zhao R., Wang H., Liu Z., Wang J. (2023). Ag Single Atoms Anchored on CeO_2_ with Interfacial Oxygen Vacancies for Efficient CO_2_ Electroreduction. ACS Appl. Mater. Interfaces.

[30] Yang X., Du C., Zhu Y., Peng H., Liu B., Cao Y., Zhang Y., Ma X., Cao C. (2022). Constructing defect-rich unconventional phase Cu_7.2_S_4_ nanotubes via microwave-induced selective etching for ultra-stable rechargeable magnesium batteries. Chem. Eng. J..

[31] Zhao Z., Li X., Wang J., Lv X., Wu H.B. (2021). CeO_2_-modified Cu electrode for efficient CO_2_ electroreduction to multi-carbon products. J. CO_2_ Util..

[32] Han Y., Wang Y., Gao W., Wang Y., Jiao L., Yuan H., Liu S. (2011). Synthesis of novel CuS with hierarchical structures and its application in lithium-ion batteries. Powder Technol..

[33] Zhang X., Yu J., Shen H.-J., Zhang L., Yang G.-X., Zhou X.-C., Feng J.-J., Wang A.-J. (2023). Reconstituting Cu^0^/Cu^+^ synergy with heterostructured CeO_2_ enabling energy-efficient bipolar hydrogen generation. Chem. Eng. J..

[34] Wu Y.-C., Huang Y.-T., Yang H.-Y. (2016). Crystallization mechanism and photocatalytic performance of vanadium-modified bismuth oxide through precipitation processes at room temperature. CrystEngComm.

[35] Zhuang T.-T., Liang Z.-Q., Seifitokaldani A., Li Y., De Luna P., Burdyny T., Che F., Meng F., Min Y., Quintero-Bermudez R. (2018). Steering post-C-C coupling selectivity enables high efficiency electroreduction of carbon dioxide to multi-carbon alcohols. Nat. Catal..

[36] Wu Z., Yu J., Wu K., Song J., Gao H., Shen H., Xia X., Lei W., Hao Q. (2022). Ultrafine CuS anchored on nitrogen and sulfur Co-doped graphene for selective CO_2_ electroreduction to formate. Appl. Surf. Sci..

[37] Zhao S., Jiang J., Zhang C., Chen F., Song Y., Tang Y. (2023). Construction of a novel double S-scheme heterojunction CeO_2_/g-C_3_N_4_/Bi_2_O_4_ for significantly boosted degradation of tetracycline: Insight into the dual charge transfer mode. Chem. Eng. J..

[38] Azenha C., Mateos-Pedrero C., Alvarez-Guerra M., Irabien A., Mendes A. (2022). Binary copper-bismuth catalysts for the electrochemical reduction of CO_2_: Study on surface properties and catalytic activity. Chem. Eng. J..

[39] Jiang B., Zhang X.-G., Jiang K., Wu D.-Y., Cai W.-B. (2018). Boosting Formate Production in Electrocatalytic CO_2_ Reduction over Wide Potential Window on Pd Surfaces. J. Am. Chem. Soc..

[40] Li S., Sha X., Gao X., Peng J. (2023). Al-Doped Octahedral Cu_2_O Nanocrystal for Electrocatalytic CO_2_ Reduction to Produce Ethylene. Int. J. Mol. Sci..

[41] Anson F.C. (1964). Application of Potentiostatic Current Integration to the Study of the Adsorption of Cobalt(III)−(Ethylenedinitrilo(tetraacetate)) on Mercury Electrodes. Anal. Chem..

[42] Yang S., Jiang M., Zhang W., Hu Y., Liang J., Wang Y., Tie Z., Jin Z. (2023). In Situ Structure Refactoring of Bismuth Nanoflowers for Highly Selective Electrochemical Reduction of CO_2_ to Formate. Adv. Funct. Mater..

[43] Liu Z., Zhang J., Yu L., Wang H., Huang X. (2022). Thermal derived bismuth nanoparticles on nitrogen-doped carbon aerogel enable selective electrochemical production of formate from CO_2_. J. CO2 Util..

[44] He C., Chen S., Long R., Song L., Xiong Y. (2020). Design of CuInS_2_ hollow nanostructures toward CO_2_ electroreduction. Sci. China Chem..

[45] Zheng X., De Luna P., García de Arquer F.P., Zhang B., Becknell N., Ross M.B., Li Y., Banis M.N., Li Y., Liu M. (2017). Sulfur-Modulated Tin Sites Enable Highly Selective Electrochemical Reduction of CO_2_ to Formate. Joule.

[46] Zhou X., Shan J., Chen L., Xia B.Y., Ling T., Duan J., Jiao Y., Zheng Y., Qiao S.-Z. (2022). Stabilizing Cu^2+^ Ions by Solid Solutions to Promote CO_2_ Electroreduction to Methane. J. Am. Chem. Soc..

[47] Ren D., Fong J., Yeo B.S. (2018). The effects of currents and potentials on the selectivities of copper toward carbon dioxide electroreduction. Nat. Commun..

[48] Wen G., Lee D.U., Ren B., Hassan F.M., Jiang G., Cano Z.P., Gostick J., Croiset E., Bai Z., Yang L. (2018). Orbital interactions in Bi-Sn bimetallic electrocatalysts for highly selective electrochemical CO_2_ reduction toward formate production. Adv. Energy Mater..

[49] Zheng Y., Vasileff A., Zhou X., Jiao Y., Jaroniec M., Qiao S.-Z. (2019). Understanding the roadmap for electrochemical reduction of CO_2_ to multi-carbon oxygenates and hydrocarbons on copper-based catalysts. J. Am. Chem. Soc..

[50] Gao Y., Guo Y., Zou Y., Liu W., Luo Y., Liu B., Zhao C. (2023). Hydrothermal Synthesis of CuS Catalysts for Electrochemical CO_2_ Reduction: Unraveling the Effect of the Sulfur Precursor. ACS Appl. Energy Mater..

[51] Tan S.M., Sofer Z., Pumera M. (2015). Sulfur poisoning of emergent and current electrocatalysts: Vulnerability of MoS_2_, and direct correlation to Pt hydrogen evolution reaction kinetics. Nanoscale.

[52] Huang Y., Deng Y., Handoko A.D., Goh G.K., Yeo B.S. (2018). Rational design of sulfur-doped copper catalysts for the selective electroreduction of carbon dioxide to formate. ChemSusChem.

[53] Wen Y., Fang N., Liu W., Yang T., Xu Y., Huang X. (2023). Cu_7_S_4_ nanosheets enriched with Cu–S bond for highly active and selective CO_2_ electroreduction to formate. J. Mater. Chem. A.

[54] Cao S., Xue Y., Chen X., Zhang C., Gao Y., Li Y. (2023). Graphdiyne/copper sulfide heterostructure for active conversion of CO_2_ to formic acid. Mater. Chem. Front..

[55] Lim J., Garcia-Esparza A.T., Lee J.W., Kang G., Shin S., Jeon S.S., Lee H. (2022). Electrodeposited Sn–Cu@Sn dendrites for selective electrochemical CO_2_ reduction to formic acid. Nanoscale.

[56] Wu D., Wang X., Fu X.-Z., Luo J.-L. (2021). Ultrasmall Bi nanoparticles confined in carbon nanosheets as highly active and durable catalysts for CO_2_ electroreduction. Appl. Catal. B Environ..

[57] Zhang L., Zhao X., Chu Z., Wang Q., Cao Y., Li J., Lei W., Cao J., Si W. (2023). Construction of Co-decorated 3D nitrogen doped-carbon nanotube/Ti_3_C_2_Tx-MXene as efficient hydrogen evolution electrocatalyst. Int. J. Hydrogen Energy.

[58] Guo D., Li X., Jiao Y., Yan H., Wu A., Yang G., Wang Y., Tian C., Fu H. (2022). A dual-active Co-CoO heterojunction coupled with Ti_3_C_2_-MXene for highly-performance overall water splitting. Nano Res..

[59] Gao S., Lin Y., Jiao X., Sun Y., Luo Q., Zhang W., Li D., Yang J., Xie Y. (2016). Partially oxidized atomic cobalt layers for carbon dioxide electroreduction to liquid fuel. Nature.

[60] Gong Q., Ding P., Xu M., Zhu X., Wang M., Deng J., Ma Q., Han N., Zhu Y., Lu J. (2019). Structural defects on converted bismuth oxide nanotubes enable highly active electrocatalysis of carbon dioxide reduction. Nat. Commun..

