# Supplementary material for: Bi/CeO2–Decorated CuS Electrocatalysts for CO2-to-Formate Conversion
Source: Molecules. 2024 Jun 21;29(13):2948. doi: 10.3390/molecules29132948 (PMC11243283; doi:10.3390/molecules29132948)
Supplement: Supplementary file 1 [file molecules-29-02948-s001.zip › molecules-2998876-supplementary.pdf]

Supporting Information

On the Role of Bi and CeO_2 for the Selective Electrochemical Reduction of CO_2 to Formate on CuS Catalysts

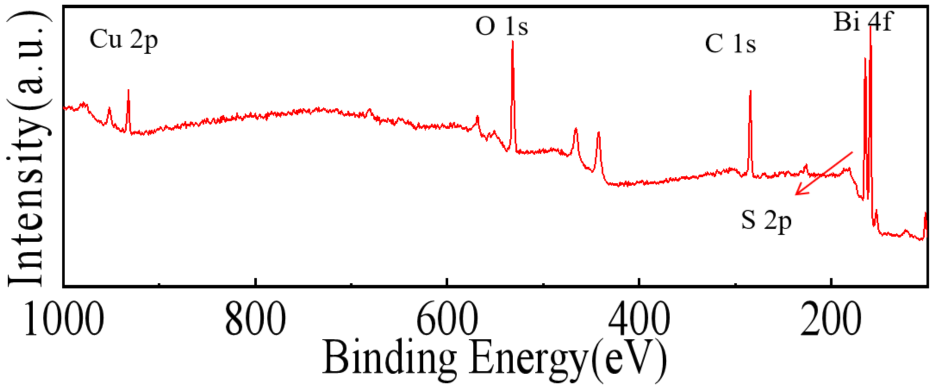

Figure S1. Survey of Bi/CeO_2/CuS

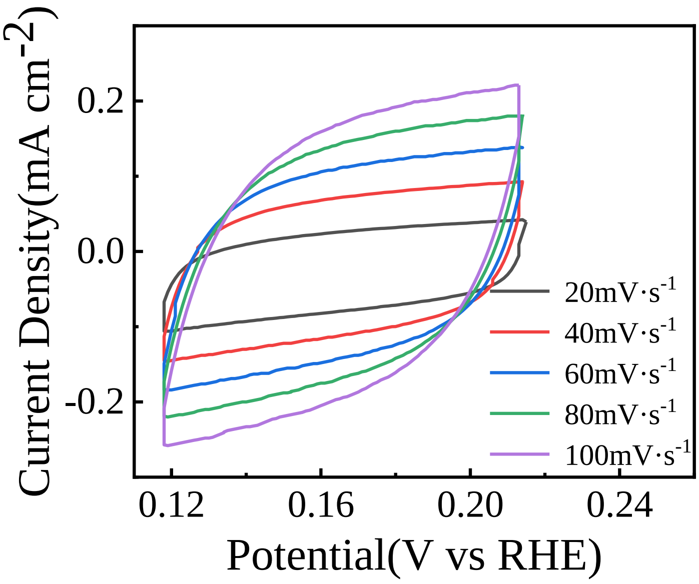

Figure S2. CV curve of CuS

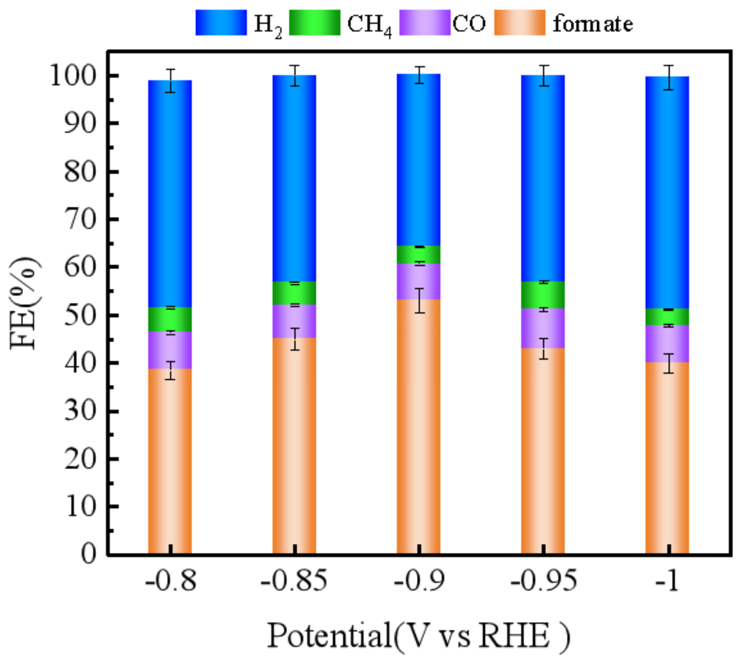

Figure S3. The FE for all products of CuS catalyst

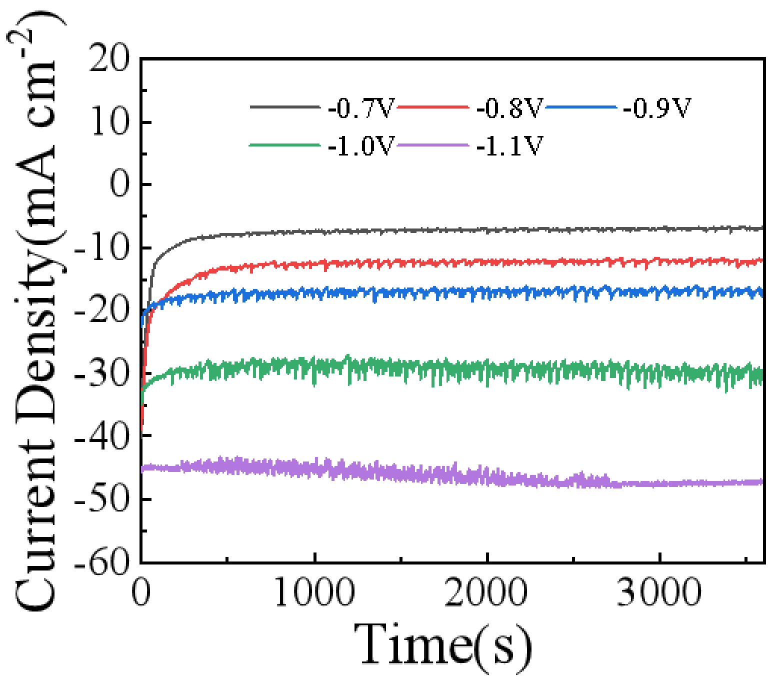

Figure S4. The current density of Bi/CeO_2/CuS at different potential

The chronoamperometry (CA) curves at various applied potentials for 1 h are shown in Figure S4, the negligible decay of current density at -1.0 V and

above indicates a stable electrocatalytic proces.

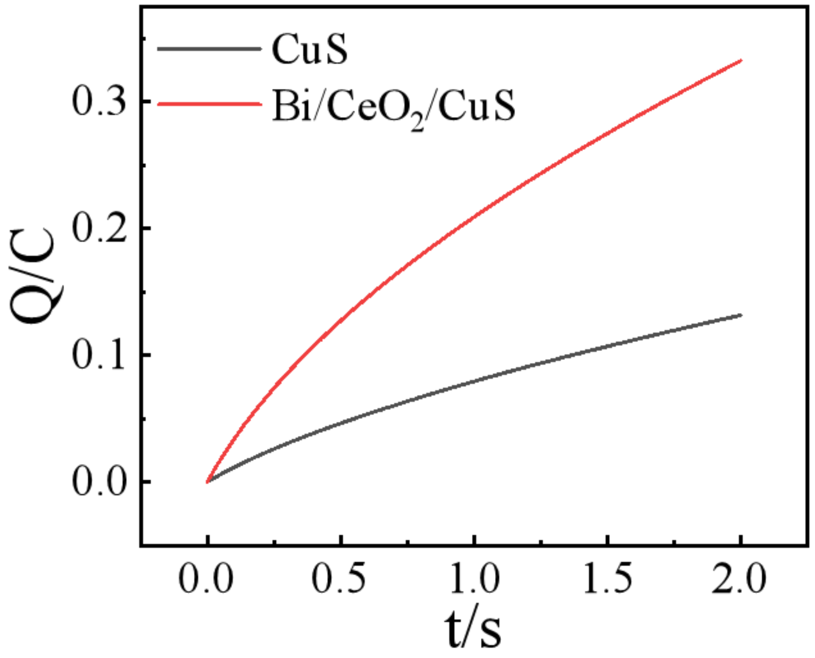

Figure S5. The effective surface area curves of different simples

Figure S6 shows the chronocoulometric curve of the CuS and $Bi/CeO_2/CuS$ in 1mmol L^{-1} $K_3[Fe(CN)_6]$ containing 0.1 mol L^{-1} KCl. According to the formula given by Anson[1].

$Q = 2nFAcD^{1/2}\ t^{1/2}/(\pi^{1/2})$

where A (cm^2) is the area of the electrode, F is the Faraday constant, D ($cm^2\ s^{-1}$) is the diffusion coefficient of the oxidized form, hexacyanoferrate(III), c (mol cm^{-3}) is the bulk concentration of the oxidized form, t (s) is the time. For 1 mM $K^3[Fe(CN)^6]$, n=1, $D=7.6\times10^{-6}$ cm^2 s^{-1}. The electroactive surface areas of the the CuS and $Bi/CeO_2/CuS$ were calculated to be 0.0132 cm^2 and 0.032 cm^2, respectively. It indicate the $Bi/CeO_2/CuS$ possess highest electroactive surface area, enhancing the electrochemical response.

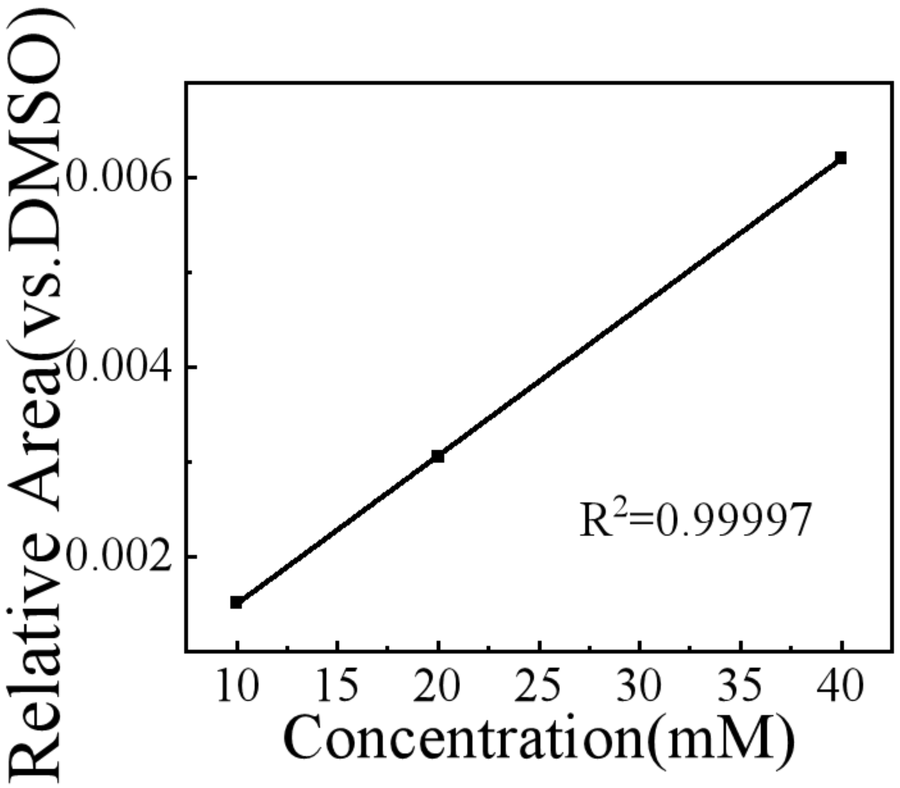

Figure S6. Standard curve of formate

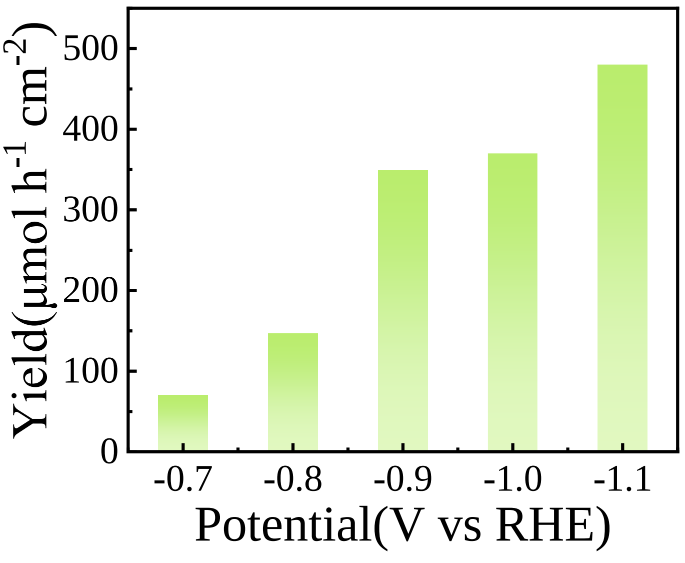

Figure S7. Formate yield of $Bi/CeO_2/CuS$

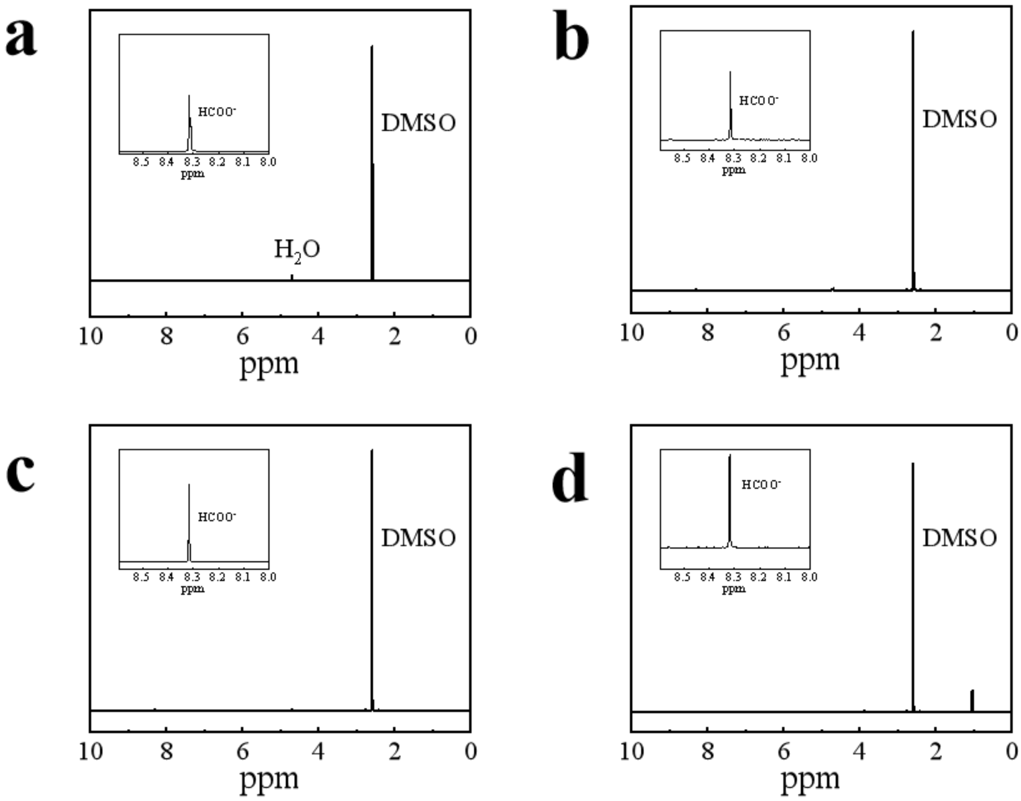

Figure S8. (a-c) The NMR of different concentration standard, (d) The NMR of $Bi/CeO_2/CuS$ at -0.9 V vs. RHE

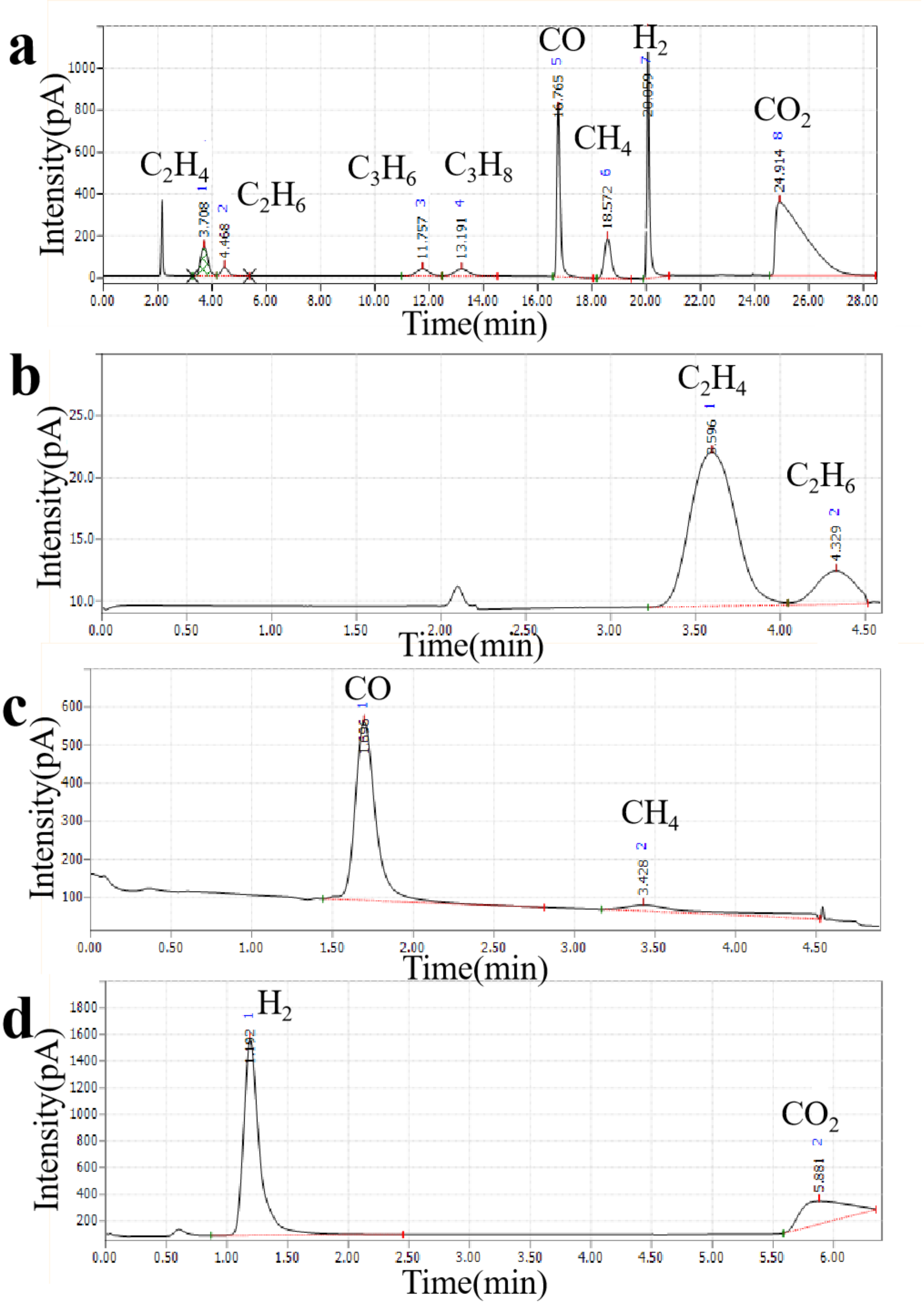

Figure S9. (a) The GC of standard, (b) The GC FID 1 image of simple, (c) The GC FID 2 image of simple, (d) The GC TCD image of simple

Reference

[1] F.C. Anson, Application of Potentiostatic Current Integration to the Study of the Adsorption of Cobalt(III)-(Ethylenedinitrilo(tetraacetate) on Mercury Electrodes, Analytical Chemistry, 36 (1964) 932-934.
